# Unraveling Impact of Hemoglobin F and A2 Levels: Correlation With Disease Severity and Treatment Response in Transfusion-Dependent Beta-Thalassemia

**DOI:** 10.7759/cureus.52002

**Published:** 2024-01-10

**Authors:** Khalid Nawaz, Sadiq Noor Khan, Aimal Bashir, Abdur Rehman, Muhammad Tariq Masood Khan, Awal Mir, Shehryar Ahmad

**Affiliations:** 1 Institute of Paramedical Sciences, Khyber Medical University, Peshawar, PAK; 2 Medical Laboratory Technology, University of Haripur, Haripur, PAK; 3 Pathology, Pak International Medical College, Peshawar, PAK; 4 Hematology, Khyber Medical University, Peshawar, PAK; 5 Health Sciences, Police Services Hospital, Directorate General Health Services, Khyber Pakhtunkhwa, Peshawar, PAK

**Keywords:** hbf induction response, disease severity parameters, hemoglobin f level, hemoglobin a2 level, transfusion-dependent β-thalassemia patient

## Abstract

Background: Fetal hemoglobin (HbF) has been reported to be associated with disease severity and treatment response to HbF-inducing therapies like Hydroxyurea and thalidomide in patients suffering from transfusion-dependent beta-thalassemia (TDT). However, the role of hemoglobin A2 (HbA2) remains less clear in TDT, therefore this study aims to determine the impact of both HbF and HbA2 levels on disease severity and treatment response.

Methodology: A prospective observational study was conducted at the Peshawar Institute of Medical Sciences and Fatimid Foundation Peshawar from May 2023 to October 2023. A total of 232 TDT-diagnosed patients were enrolled using a convenient sampling technique, whereas coinheritance of beta-thalassemia with other hemoglobinopathies was excluded.

Result: This study reveals a significant impact of HbF on disease severity (p<0.05) but finds no substantial correlation (p>0.05) between HbA2 levels and disease severity. Additionally, HbF and HbA2 levels exhibit no association with treatment response categories in patients receiving HbF induction therapy, and various mutations do not significantly alter HbF and HbA2 levels or disease severity parameters in TDT patients.

Conclusion: The study established a significant association between HbF and disease severity. However, regarding treatment response, neither HbF nor HbA2 levels impact response categories. Combinatorial treatment with hydroxyurea and thalidomide showed superior efficacy compared to monotherapy. A larger sample size and extended follow-up are recommended to further explore the impact of HbF, HbA2, and various mutations on disease severity and treatment response.

## Introduction

Transfusion-dependent beta-thalassemia (TDT) is a common hereditary disease caused by mutations in the gene encoding β-chains of hemoglobin (Hb) which leads to the reduced or absent synthesis of the β-globin chain in Hb tetramers. [1] These patients require continuous blood transfusions or bone marrow transplantation for their survival [2]. According to the World Health Organization (WHO), about 25,500 neonates globally are born with TDT each year [3]. Pakistan is considered one of the highest prevalence countries of TDT patients with an estimated 50,000 to 100,000 patients suffering, and approximately 6000 newborns affected yearly [4,5]. It has been established that lifelong blood transfusion and iron chelation therapy (ICT) impose a significant burden on both children and their families, especially in terms of risk for transfusion-transmitted infections (TTI), and a serious economic burden on the healthcare system [4,6,7]. The comprehensive treatment cost for 60,000 TDT patients in Pakistan is about 7.8 billion rupees annually. This covers expenses for supportive treatments like blood transfusions (Rs. 30,000) and ICT (Rs. 150,000 per child per year). Hematopoietic stem cell transplantation (HSCT), the disease's only cure, is only affordable for a limited number of individuals [7].

Infants with thalassemia major show poor growth, increasing pallor, feeding difficulties, diarrhea, irritability, recurrent fevers, and abdominal enlargement may occur due to hepatosplenomegaly [8]. The clinical severity of the disease depends on molecular mutations. Over 250 mutations recently in the β-globin gene have been identified. The imbalance between Alpha (α) and Beta (β) chains results in the accumulation of insoluble alpha chains, leading to hemolysis in peripheral circulation and infective erythropoiesis in the bone marrow. The degree of imbalance is directly proportional to disease severity. Hemolysis and ineffective erythropoiesis culminate in severe anemia, along with erythroid hyperplasia, bone marrow expansion, extramedullary hematopoiesis, and iron overload. The marrow expansion contributes to skeletal abnormalities, notably affecting facial bones and causing frontal bossing and maxillary protrusion. Biochemical signaling associated with marrow expansion, particularly involving the bone morphogenetic protein (BMP) pathway, hinders hepcidin production, consequently promoting excessive iron absorption (iron overload) [9-11]. While genetic factors disrupting the equilibrium of globin chain synthesis are primary genetic modifiers that encompass the type of β-thalassemia mutation (β0/β+/β++), secondary genetic modifiers include concurrent α-thalassemia and polymorphisms linked to HbF levels. Tertiary loci, while not directly influencing globin production, might have the potential to modulate disease complications in diverse manners [10,12,13].

Fetal hemoglobin (HbF) represents a critical determinant of disease severity in individuals afflicted with TDT [13]. Three prominent HbF quantitative trait loci (QTLs), responsible for 20-50% of HbF variation, have been revealed so far including B-cell lymphoma/leukemia (*BCL11A*) gene, the HBS1L-MYB intergenic region and Krüppel-like factor 1 (KLF) [13,14]. The induction of HbF production stands as a novel therapeutic objective for patients with hemoglobinopathies. This induced elevation of γ-globin chains serves to counterbalance the deleterious α-globin chains, consequently ameliorating anemia and restricting transfusion dependency. Currently, various pharmacological agents have been assessed for their ability to induce HbF. Among these, hydroxyurea (HU) and thalidomide have emerged as notable HbF inducers, leading to a significant increase in HbF concentrations in TDT patients [1,15]. Studies have also shown an alarmingly high mortality rate in beta-thalassemic patients with increased ferritin levels [2]. ICT can decrease the morbidity associated with iron toxicity. Injectable deferoxamine is recommended as a long-term iron chelator for β-thalassemia and other hemochromatosis conditions. It effectively reduces hepatic iron overload and serum ferritin levels, thereby contributing to increased longevity [16]. Additional treatment modalities for TDT comprise HSCT, the emerging field of gene therapy, and erythroid maturation agents [1].

TDT is still a trouble for the healthcare system and families, particularly in developing countries where the disease is prevalent. To address this issue, this study was conducted to bridge the knowledge gap by investigating the correlation of HbF and HbA2 levels with the disease severity in TDT patients because, to the best of our knowledge, there is scarce literature on the explicit role of HbA2 with severity in TDT. The study also aimed to uncover the role of HbF and HbA2 in treatment response. Furthermore, we compared the parameters of patients who were on regular blood transfusion and ICT alone with patients who were taking HbF inducer drugs. The hope is that this study will contribute unexplored insights into the disease and its treatment, and ultimately will have a positive impact on the scientific community.

## Materials and methods

This was a prospective observational study conducted at the Peshawar Institute of Medical Sciences in Peshawar Pakistan. Ethical committee Institute of Paramedical Sciences Khyber Medical University, Peshawar issued approval DIR/KMU-EB/ED/000879. Informed consent was taken from the patient’s parents and research proforma was filled out including demographic information and clinical data was collected under the ethical principles established in the Helsinki Declaration (World Medical Association, 2013) for human samples. Patient’s privacy and confidentiality were assured at every step.

A total of 232 TDT-diagnosed patients were included. Patients with coinheritance of β-thalassemia with other hemoglobinopathies, and who received partial or irregular treatment were excluded. The study spanned over a period of six months, from May 2023 to October 2023. The study population was divided into two groups. Group I included patients receiving regular red cell concentrate (RCC) transfusions with ICT. In Group II, patients received proper HbF inducers such as HU and/or thalidomide and RCC transfusions along with iron chelation whenever required. The treatments were classified into three categories: HU alone, thalidomide alone, and a combination of both. The treatment response in Group II was assessed based on predefined criteria as shown in Table 1. As a criterion, treatment response was evaluated 60 days after the initiation of treatment, with a focus on Hb levels.

**Table 1:** Predefined criteria for disease severity parameters and treatment responses in transfusion-dependent beta-thalassemia utilized in this prospective observational study g/dL: gram per deciliter; ng/mL: nanogram per milliliter; cm: centimeter

Research Parameters	Criteria perimeter/treatment duration	Sub-groups	Cutoff Range
Anemia	Hemoglobin level (g/dL)	Normal	>12
		Mild	10-12
		Moderate	10-7
		Severe	<7
Iron Overload	Serum ferritin level (ng/mL)	Normal	< 1000
		Mild	1000-2000
		Moderate	2000-5000
		Severe	> 5000
Spleen Size	Ultrasound-based spleen size (cm)	Normal	0
		Mild	1-2
		Moderate	2-5
		Severe	> 5
Liver Size	Ultrasound-based liver size (cm)	Normal	0
		Mild	1-2
		Moderate	2-5
		Severe	> 5
Treatment Response	60 days post-treatment Hb levels (g/dL)	Excellent	>9
		Good	7-8.9
		Partial	6-6.9
		No Response	<6

For disease severity, we chose five parameters, i.e., anemia, iron overload, transfusion frequency, splenomegaly, and hepatomegaly. A venous blood sample of about 03 mL was collected in two each K3 EDTA vacutainer tube (BD, China) for complete blood count (CBC), hemoglobin electrophoresis, and beta gene mutation analysis by multiplex PCR. Two mL was also collected in a non-additive vacutainer (BD, China) for ferritin, bilirubin, and alanine aminotransferase (ALT) analysis. CBC test was analyzed using an automated hematology analyzer (XN-1000, Sysmex, Japan) while Hb electrophoresis was performed using a capillary electrophoresis analyzer (Sebia, Capillary 2, France). All chemical pathology parameters were analyzed on Cobas chemistry analyzer (c 111, Roche, Japan). Data was analyzed using SPSS version 22. For quantitative variables mean and standard deviation were determined, whereas for qualitative variables frequencies and percentages were calculated. Regarding inferential statistics, one-way analysis of variance (ANOVA) was used to examine the correlation between HbF and HbA2 levels with disease severity, and treatment response. Additionally, we conducted the Chi-square test to explore the relationship between genotyping and disease severity parameters. The results were displayed in the form of graphs and tables. A P-value of less than 0.05 was considered a statistically significant finding.

## Results

We had a total of 232 subjects, Group I (n=100) and Group II (n=132), among which 140 (60.34%) were males and 92 (39.65%) were females. The mean age of Group-1 patients was 13.5±7.67 years, while the average age for Group-II treatment response patients was 5.8±4.7 years, respectively.

Comparative analysis of group I and II parameters

Group I comprised 100 patients having a mean HbF level of 82.58% and HbA2 level of 4.02%. In contrast, Group II consisted of 132 patients with a mean HbF level of 82.52% and an HbA2 level of 2.62%. There is a big difference between HbA2 levels among the groups. The initial age in months at which patients in both categories received their first blood transfusion was 8.64±10.88 and 9.4±9.2 months, respectively. Furthermore, Group I patients have a mean Hb level of 7.17±1.79 g/dL whereas Group II maintained a mean Hb level of 8.35±1.83 g/dL after receiving HbF induction therapy and infrequent red blood cell transfusion. Additionally, Group I exhibited an average transfusion frequency of 18.2±8.9 days, while Group II had an average transfusion interval of 68.41±189.78 days. Consolidated pertinent parameters of the study can be seen in Tables 2-3

**Table 2:** Comparison of disease severity parameters between patients receiving regular RBC transfusion with iron chelation (Group 1) and those who received a specific HbF inducer with infrequent RBC transfusion and iron chelation (Group II) The table distinctly illustrates differences between patients exclusively undergoing regular transfusion combined with ICT and those receiving comprehensive treatment. Notably, patients reliant solely on transfusion exhibited more severe disease parameters. Further key observations include a 72% increase in transfusion frequency over time among the transfusion-only group, in stark contrast to a substantial 78.1% reduction in transfusion frequency in patients receiving proper treatment. The prevalence of splenectomies patients was also notably higher among those exclusively receiving transfusion, standing at 17%, compared to a considerably lower 5.3% among treatment recipients. Post-complications were more frequent among transfusion patients, including hepatitis C virus (HCV) (25%), one case of HbsAg, and four cases of skin reactions (blisters, body aches, rashes). In contrast, treatment patients reported minor consequences, such as temporary body pain in 5.3% of cases and skin complications (blisters, body aches, rashes) in 9.1% of cases. However, it is noteworthy that a majority of both transfusion (69.4%) and treatment (84.4%) patients did not experience any complications in our study. HCV: hepatitis C virus; HbF: fetal hemoglobin; RCC: red cell concentrate

Disease Severity Parameters			Group-I Numbers (%)	Group-II Numbers (%)
Anemia	Normal		0 (0)	4 (3)
	Mild		8 (8)	23 (17.4)
	Moderate		45 (45)	72 (54.5)
	Severe		47 (47)	33 (25)
Transfusion Frequency	Average RCC unit transfusion in 90 days		4.8	1.3
Spleen Size	Normal		4 (4)	40 (30.3)
	Mild		28 (28)	41 (31.1)
	Moderate		46 (46)	23 (17.4)
	Severe		5 (5)	21 (15.9)
Splenectomy	Yes		17 (17)	7 (5.3)
	No		83 (83)	125 (94.7)
Hepatomegaly	Normal		4 (4)	14 (10.6)
	Mild		32 (32)	58 (43.9)
	Moderate		60 (60)	36 (27.3)
	Severe		4 (4)	24 (18.2)
Iron Overload	Normal iron level		6 (6)	28 (42.4)
	Mild		8 (8)	15 (22.7)
	Moderate		46 (46)	14 (21.2)
	Severe		40 (40)	9 (13.6)
Complications	No		68 (69.4)	112 (84.4)
	Yes	HCV	25 (25.1)	0 (0)
		HBsAg	1 (1)	0 (0)
		Skin reactions	4 (4.5)	13 (9.1)
		Bone pain	0 (0)	7 (5.3)

**Table 3:** A comparison between the hematological and biochemical parameters of Group I (regular RBC transfusion with iron chelation) and Group II (HbF induction with infrequent RBC transfusion and iron chelation) Hb: hemoglobin; RBC: total RBC count; MCV: mean corpuscular volume; MCH: mean corpuscular hemoglobin; MCHC: mean corpuscular hemoglobin concentration; TLC: total leukocytes count; ALT: alanine transaminase; HbF: hemoglobin F; HbA2: hemoglobin A2; HbA1: hemoglobin A1; g/dL: gram per deciliter; 10^12 /L: trillion per liter; fL: femtoliter; pg: picogram; 10^9/L: billion/L; ng/mL: nanogram per milliliter; u/I: international unit; mg/dL: milligram per deciliter; p-value <0.005= statistically significant value.

Hemato-Biochemical Parameters	Normal Values	Group-I (Mean ± SD)	Group II (Mean ± SD)	P-Value
Hb (g/dL)	12-16	7.17±1.179	8.35±1.83	<0.001
RBC (10^12 /L)	4.5-5.5	2.84±0.72	3.68±2.81	
HCT (%)	40-54	22.08±5.87	25.30±5.87	
MCV (fL)	80-100	77.53±7.93	75.82±7.81	
MCH (pg)	27-32	25.23±2.18	25.45±4.85	
MCHC (g/dL)	31-35	32.60±2.78	32.79±3.51	
TLC (10˄9/L)	4-11	8.34±7.23	12.63±13.85	
Platelets (10^9/L)	150-450	282.13±191.82	313.17±157	
Ferritin (ng/mL)	18-220	4827±3074.85	2005.26±2115.48	
ALT (u/I)	10-45	101.17±233.3	49.52±54.94	
Total Bilirubin (mg/dL)	<1.0	1.41±1.11	1.35±1.32	
HbF (%)	0.8-2	83.73±21	82.55±22.27	
HbA2 (%)	2-3.5	4.02±8.42	2.65±1.34	
HbA1 (%)	95-98	12.3±19.28	12.71±20.3	

Association of HbF and HbA2 levels with disease severity

To examine the association between HbF and HbA2 levels and the severity of the disease. We conducted a one-way ANOVA that included both Group-I and Group-II disease severity parameters. The obtained p-value for HbF showed statistical significance (p<0.05), suggesting a relationship between HbF levels and disease severity. More specifically, higher levels of HbF in patients with TDT were associated with a less severe manifestation of the condition. However, for HbA2 levels, we found no statistically significant correlation (p>0.05) with disease severity.

HbF and HbA2 *Correlation with Treatment Response*

We examined the relationship between treatment response and hemoglobin F and hemoglobin A2 levels related to patients receiving hydroxyurea, thalidomide, or a combination of the two for hemoglobin induction therapy. The non-significant p-values exceeding 0.05 for both HbF and HbA2 indicate that the levels of HbF and HbA2 in patients diagnosed with TDT do not appear to have an impact on the classification of treatment response into categories like excellent, good, partial, or no response.

Determination of various mutations effects on levels of HbF and HbA2 and disease severity

**Figure 1:**
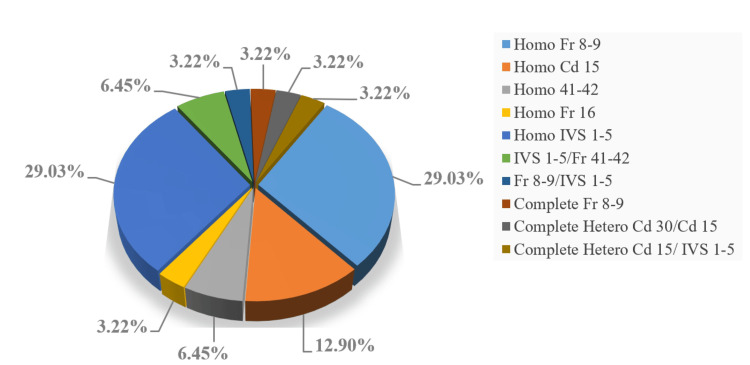
Types of mutations identified in 31 patients with transfusion-dependent beta-thalassemia. Among these, Homo Fr 8-9 and Homo IVS 1-5 were more common, accounting for 29.03% each, as observed in the current prospective study.

We analyzed mutation data from 31 patients, identifying a spectrum of 11 distinct mutations within the beta genes shown in Figure 1. Employing one-way ANOVA, we sought to discern any potential correlation between various genotypes and HbF as well as HbA2 levels. Notably, the resultant p-values did not yield significant results (p>0.05) for either HbF or HbA2. This leads us to reiterate that mutations do not exert a discernible influence on the levels of HbF and HbA2 in TDT. Furthermore, to elucidate the impact of diverse mutation types on disease severity, we conducted a Chi-square test. Similarly, the resulting p-value (>0.05) did not attain statistical significance. We might say that mutations also do not appear to play a substantial role in disease severity. Nevertheless, we propose that a larger sample size in mutation studies may uncover potential correlations that could remain undetected with smaller sample sizes.

Evaluation of treatment responses

**Figure 2:**
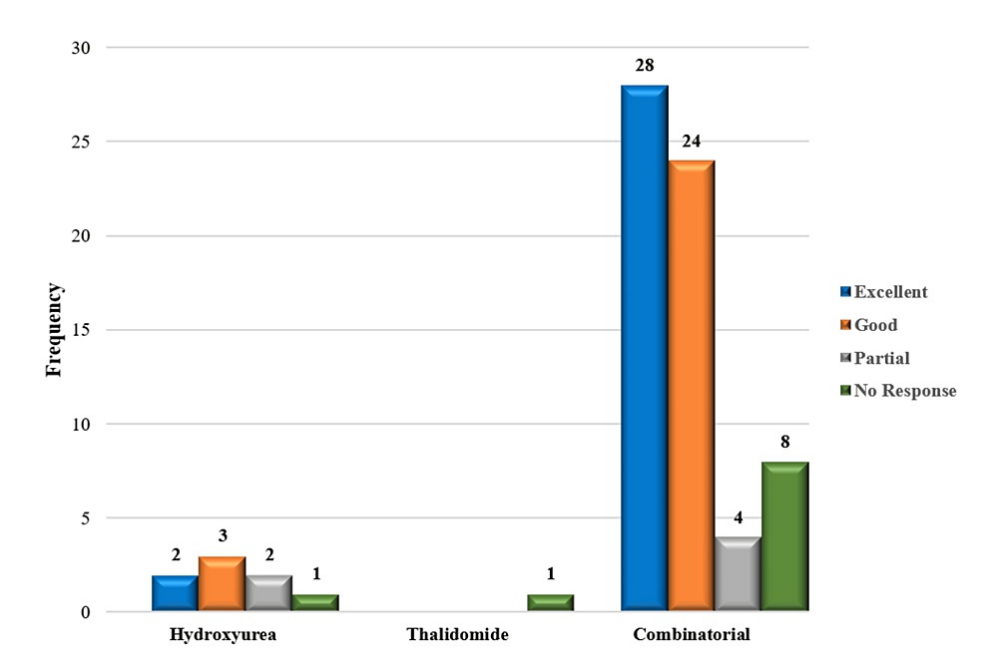
Treatment response of patients after HbF-inducing therapy without transfusion. HbF: fetal hemoglobin

**Figure 3:**
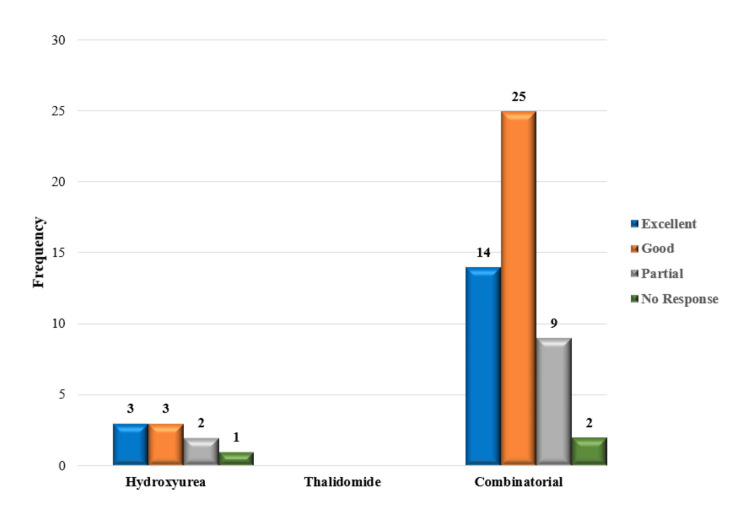
Treatment response of patients after HbF-inducing therapy with periodic transfusion. HbF: fetal hemoglobin

Of the 132 patients that were being studied, 114 patients (86.4%) were receiving HU and thalidomide as part of a combination therapy. However, a smaller group of patients 17 (12.9%) were the only receiving HU therapy, while one patient was prescribed thalidomide alone. Based on post-treatment Hb levels and transfusion status, our study examined response patterns in patients (n = 132) classified as belonging to Group II. Thirteen patients (13%) required transfusion support to obtain an excellent response, while 30 individuals (22.7%) did not require one in this group. Additionally, transfusions enhanced the good response in 28 patients (21.4%), while no transfusion was necessary for the good response in 27 patients (20.5%). Transfusions were given to 11 patients (8.4%) with partial responses and not to six patients (4.5%) with no response. Notably, 10 patients (7.6%) were non-responders without transfusion, and three patients (2.3%) showed no reaction despite getting blood transfusions. Additional information is provided in Table 4 and Figures 2-3.

**Table 4:** Comparative analysis of hematological and biochemical parameters before HbF induction (pre-treatment) and after HbF induction (post-treatment) patients Hb: hemoglobin; RBC: total RBC count; MCV: mean corpuscular volume; MCH: mean corpuscular hemoglobin; MCHC: mean corpuscular hemoglobin concentration; TLC: total leukocytes count; ALT: alanine transaminase; g/dL: gram per deciliter; 10^12 /L: trillion per liter; fL: femtoliter; pg: picogram; 10^9/L: billion/L: ng/mL: nanogram per milliliter; u/I: international unit; mg/dL: milligram per deciliter.  p-value <0.005= statistically significant value.

Hemato-Biochemical Parameters	Normal Values	Group B (Mean ± SD)		P-Value
		Pre-Treatment	Post-Treatment	
Hb (g/dL)	12-16	8.63±2.28	8.35±1.83	0.195
RBC (10^12 /L)	3.5-5.5	3.45±0.92	3.68±2.81	0.358
HCT (%)	40-54	26.15±8.10	25.30±5.87	0.268
MCV (fL)	80-100	75.63±10.25	75.82±7.81	0.828
MCH (pg)	27-32	25.17±3.82	25.45±4.85	0.583
MCHC (g/dL)	31-35	33±3.4	32.8±2.51	0.583
TLC (10^9/L)	4-11	11.57±8.62	12.63±13.85	0.386
Platelets	150-450	298.8±155.35	313.7±157	0.305
Ferritin (ng/mL)	18-220	2880±2637	2005±2115	<0.001
ALT (u/I)	10-45	53.9±67.19	49.5±55	0.4.38
Total Bilirubin (mg/dL)	<1.0	1.46±1.97	1.35±1.32	0.541

## Discussion

Several studies have explored the relationship between HbF and disease severity. The coexistence of hereditary persistence of HbF in patients with TDT mitigates disease severity, leading to several patients becoming non-transfusion-dependent [17]. Another study mentioned the identification of genetic variants that modify HbF production, in conjunction with the α globin genotype, offering predictive insights into disease severity in β thalassemia [18]. Additionally, it has been established that affected patients do not develop anemia until the fetal (γ) globin genes are developmentally silenced. Patients with persistently high levels of fetal globin typically exhibit less severe anemia, and milder clinical syndromes, and often do not require blood transfusions [19]. While many studies elucidate the role of elevated HbA2 in beta-thalassemia trait (βTT), less literature is available on addressing the role of HbA2 in disease severity in TDT [20-22]. Consequently, the principal objectives of our study were to ascertain the association between HbF and HbA2 levels on disease severity in TDT. As a result, we identified a significant association between HbF levels and disease severity in TDT patients whereas no significant association was observed between HbA2 levels and disease severity in the same population. However, we found a large difference in HbA2 levels among Groups I &II. Furthermore, our study is in concordant agreement with the findings of a study by Ansari et al., which reveals an insignificant association of HbF and HbA2 levels with treatment response [23].

Another cross-sectional study was conducted on 150 patients in Iraq who were undergoing regular RBC transfusion and ICT. The median age of the participants was 13 years (range: 1-35 years), with only 2.0% aged 30 years or older. The age at diagnosis, expressed in months, was six (2.5-24.0). The mean pre-transfusion Hb level stood at 8.6±1.0 g/dL, with 38.7% of patients maintaining levels ≥ 9.0 g/dL, 23.3% falling below 8.0 g/dL, and 2.7% surpassing 10.5 g/dL. The median serum ferritin concentration was 2762 µg/L. All patients were on regular transfusions, while 94.7% were concurrently undergoing chelation therapy, and 38.0% had undergone splenectomy. Detectable HCV antibodies were observed in 35.3% of cases, whereas neither HIV antibodies nor hepatitis B surface antigens were detectable in any of the subjects [24]. Similarly in our study, we examined 100 patients for only RBC transfusion and ICT with an average age of 13.5 years (range: 0.2-36 years). The initial age in months at which patients received their first blood transfusion was 9.4 months. These patients maintained a mean Hb level of ≥7.17 g/dL within a range of 3-10 g/dL, exhibiting an average transfusion frequency of 18.2 days. For transfusion complications, we reported 25% HCV cases, one case of HBsAg, four cases of skin reactions (blisters, body aches, rashes), and none of HIV. The prevalence of splenectomies patients was standing at 17% and our mean ferritin level (4827 ng/mL) was elevated as compared to the Iraq study.

HSCT, a curative treatment, is not a viable option for every patient. Hence, there is a need for an alternative treatment capable of alleviating symptoms associated with transfusion dependence and therefore iron overload [25]. HU and thalidomide, identified as potent Hb inducers, have been subjected to numerous trials, all of which have demonstrated their effectiveness in increasing Hb levels and reducing transfusion requirements in patients with TDT, whether administered individually or in combination [17,23-27]. Our findings align with the aforementioned studies, as we observed an overall response rate of 90.1% to treatment (12.9% with HU alone, 0.8% with thalidomide alone, and 86.4% with the combination). Conversely, only 9.9% of patients (1.52% with HU alone, and 7.58% with the combination) exhibited no response, either with or without blood transfusion.

Ansari et al. in their prior research, explored the influence of HU and various mutations on response categories in individuals with TDT, revealing a positive correlation between genotypes and HU response [28]. Surprisingly, in their recent experiment of the combination therapy of HU and thalidomide for TDT patients, the success of this combination response was not linked to any specific genetic mutation [23]. In our study, we directed our attention to examining the impact of a diverse spectrum of mutations in TDT patients on the levels of HbF and HbA2, as well as on disease severity parameters. As a result, we did not identify a significant correlation in these relationships. Nevertheless, we strongly advocate for the inclusion of larger sample sizes in mutation studies, as this may reveal potential correlations that might remain elusive with smaller sample sizes.

The present study has limitations, as it did not investigate the correlation between HbF and HbA2 levels and patient mortality or survival rates. Furthermore, the study did not ascertain the real-time induction of HbF (the degree of HbF level increases) in relation to the rise in total Hb levels. This could be addressed through single-cell real-time HbF and total single-cell Hb quantifications, providing a more comprehensive understanding of the dynamics involved.

## Conclusions

The study established a significant association between HbF and disease severity. However, regarding treatment response, neither HbF nor HbA2 levels impact response categories. Combinatorial treatment with HU and thalidomide showed superior efficacy compared to monotherapy. A larger sample size and extended follow-up are recommended to further explore the impact of HbF, HbA2, and various mutations on disease severity and treatment response.
